# 2-(Thio­phen-2-yl)-1-(thio­phen-2-ylmeth­yl)-1*H*-benzimidazole

**DOI:** 10.1107/S1600536811055103

**Published:** 2012-01-18

**Authors:** David K. Geiger, H. Cristina Geiger, Leo Williams, Bruce C. Noll

**Affiliations:** a Department of Chemistry, State University of New York-College at Geneseo, 1 College Circle, Geneseo, NY 14454, USA; b Chemical Crystallography, Bruker AXS Inc., 5465 East Cheryl Parkway, Madison, Wisconsin, 53711, USA, and 7517 East Pass, Madison, Wisconsin, 53719, USA

## Abstract

In the title compound, C_16_H_12_N_2_S_2_, the thio­phene groups are rotationally disordered over two sets of sites, by approximately 180°, with occupancy ratios of 0.916 (2):0.084 (2) and 0.903 (2):0.097 (2). The major components of the thio­phene and methyl­ene substituted thio­phene rings are canted by 24.06 (12) and 85.07 (10)°, respectively, from the benzimidazole ring system plane and the dihedral angle between the major component thio­phene ring planes is 84.90 (14)°. In the crystal, there is a weak C—H⋯N hydrogen bond which links mol­ecules into chains.

## Related literature

For a discussion of the rearrangement of 1,2-diimino­benzene species to form benzimidazoles, see: Smith & Ho (1971 ▶). See Varala *et al.* (2007 ▶) for examples of proline-catalysed 1,2-disubstituted benzimidazole syntheses. Reich *et al.* (2004 ▶) provide examples of inter­molecular aldimine coupling. For other syntheses of substituted benzimidazoles, see: Grimmett (1997 ▶); Bahrami *et al.* (2007 ▶); Du & Wang (2007 ▶). For the biological activity of benzimidazole derivatives, see: López-Rodríguez *et al.* (1999 ▶); Horton *et al.* (2003 ▶).
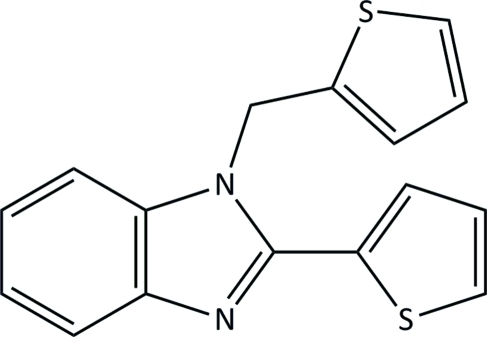


## Experimental

### 

#### Crystal data

C_16_H_12_N_2_S_2_
*M* *_r_* = 296.40
Monoclinic, 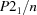

*a* = 8.9859 (13) Å
*b* = 9.1601 (11) Å
*c* = 17.476 (3) Å
β = 93.629 (5)°
*V* = 1435.6 (3) Å^3^
*Z* = 4
Mo *K*α radiation
μ = 0.36 mm^−1^
*T* = 300 K
0.80 × 0.30 × 0.10 mm

#### Data collection

Bruker SMART X2S benchtop diffractometer
Absorption correction: multi-scan (*SADABS*; Bruker, 2008 ▶) *T*_min_ = 0.761, *T*_max_ = 0.965
8857 measured reflections
2527 independent reflections
1942 reflections with *I* > 2σ(*I*)
*R*_int_ = 0.033

#### Refinement

*R*[*F* ^2^ > 2σ(*F* ^2^)] = 0.040
*wR*(*F* ^2^) = 0.103
*S* = 1.05
2527 reflections
210 parameters
26 restraints
H atoms treated by a mixture of independent and constrained refinement
Δρ_max_ = 0.18 e Å^−3^
Δρ_min_ = −0.25 e Å^−3^

### 

Data collection: *APEX2* (Bruker, 2010 ▶); cell refinement: *SAINT* (Bruker, 2009 ▶); data reduction: *SAINT*; program(s) used to solve structure: *SHELXS97* (Sheldrick, 2008 ▶); program(s) used to refine structure: *SHELXL97* (Sheldrick, 2008 ▶); molecular graphics: *XSHELL* (Bruker, 2004 ▶); software used to prepare material for publication: *SHELXL97*.

## Supplementary Material

Crystal structure: contains datablock(s) I, global. DOI: 10.1107/S1600536811055103/lh5359sup1.cif

Supplementary material file. DOI: 10.1107/S1600536811055103/lh5359Isup2.mol

Structure factors: contains datablock(s) I. DOI: 10.1107/S1600536811055103/lh5359Isup3.hkl

Supplementary material file. DOI: 10.1107/S1600536811055103/lh5359Isup4.mol

Supplementary material file. DOI: 10.1107/S1600536811055103/lh5359Isup5.cml

Additional supplementary materials:  crystallographic information; 3D view; checkCIF report

## Figures and Tables

**Table 1:** Hydrogen-bond geometry (Å, °)

*D*—H⋯*A*	*D*—H	H⋯*A*	*D*⋯*A*	*D*—H⋯*A*
C16—H16⋯N2^i^	0.93	2.54	3.445 (4)	166

Symmetry code: (i) 
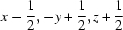
.

**Fig. 1.**
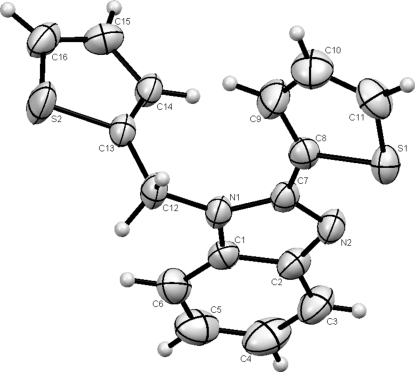
Perspective view of the title compound with displacement ellipsoids of non-hydrogen atoms drawn at the 50% probability level. The disorder is not shown.

**Fig. 2.**
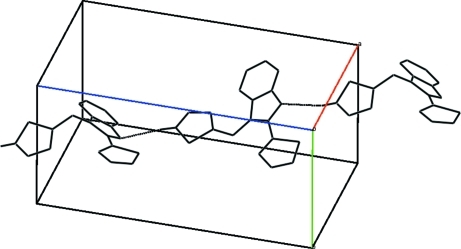
View of the C—H···N intermolecular interactions leading to chain formation.

**Fig. 3.**
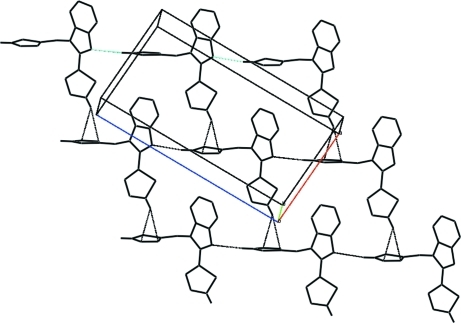
View of layer formed by joining of chains by bifurcated C—H···C intermolecular contacts.

**Fig. 4.**
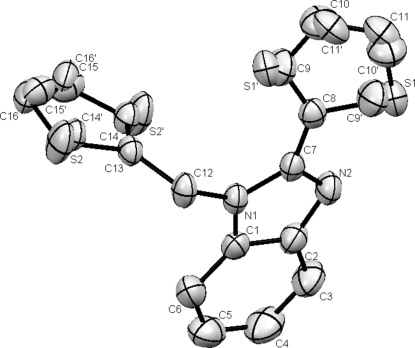
The title molecule with displacement ellipsoids of non-hydrogen atoms drawn at the 50% probability level. Major and minor components of the disordered thiophene groups are shown. Hydrogen atoms have been ommitted for clarity.

## supplementary crystallographic information

### Comment

Benzimidazole derivatives are of interest because of their pharmacological uses. Examples include inhibitors of serotonin activated neurotransmission (López-Rodríguez *et al.*, 1999) and antiviral agents (Varala *et al.*, 2007). They have also found use as antiarrhythmic, antihistamine, antiulcer, anticancer, fungicidal, anthelmintical drugs (Horton *et al.*, 2003).

Numerous methods are available for the synthesis of substituted benzimidazoles (Grimmett, 1997). One pot procedures for the synthesis of 2-aryl substituted benzimidazoles involving the condensation of 1,2-diaminobenzene with aldehydes employing hydrogen peroxide and ceric ammonium nitrate (Bahrami *et al.*, 2007) or hypervalent iodine (Du & Wang, 2007) as oxidizing agents have been reported. Syntheses of 1-arylmethyl-2-aryl substituted benzimidazoles *via* the proline catalyzed condensation of 1,2-diaminobenzene derivatives with aryl aldehydes have also been reported (Varala *et al.*, 2007). Attempts to prepare *N*,*N*'-dibenzal-*o*-phenylenediamine *via* the condensation of 1,2-diaminobenzene and benzaldehyde lead to presumed rearrangement of the initially formed Schiff base yielding 1-benzyl-2-phenylbenzimidazole (Smith & Ho, 1971).

Our efforts have focused on the preparation of benzimidazole analogues which have substituents capable of binding metals. Toward that end, we have prepared 1-(thiophene-2-methyl)-2-(2-thiophene)benzimidazole, TMTB, from a reaction of 1,2-diaminobenzene with 2-thiophenecarboxaldehyde.

Figure 1 shows a perspective view of TMTB with the atom-labeling scheme. Only the major components of the disordered thiophene groups are displayed. Close C—H16···N2 intermolecular interactions (2.535 Å, 165.8°) result in chains of TMTB as shown in Figure 2. Bifurcated C—H11···C14 and C15 intermolecular contacts (2.843 Å and 2.848 Å) join the chains to form a layer network (see Figure 3). Figure 4 is a perspective view of the compound showing both major and minor components of the disordered thiophene groups.

The structure exhibits the expected planar benzimidazole moiety (maximum deviation 0.0076(0.0014) Å, N1). The major components of the disordered thiophene and methylthiophene rings are canted 24.06 (12) ° and 85.07 (10) °, respectively, from the benzimidazole plane, with a thiophene-methylthiophene dihedral angle of 84.90 (14) °. Together, the thiophene groups provide a structure with the potential to behave as a bidentate ligand employing the sulfur atoms. We are exploring the coordination chemistry of TMTB.

### Experimental

The title compound was prepared by the reaction of two equivalents of 2-thiophene with 1,2-diaminobenzene in the presence of a catalytic amount of aluminium trichloride under nitrogen in dichloromethane. The reaction mixture was refluxed for 8 h,filtered and the solvent removed by rotary evaporation. The crude product was purified by column chromatography (silica gel)using 20%(*v*/*v*) ethylacetate in hexanes.

The purified product was characterized *via*^1^H and ^13^C NMR spectroscopies. ^1^H NMR spectrum (DMSO-*d_6_*, 400 MHz, p.p.m.): 8.14 (2*H*, m), 7.98 (1 H, m), 7.50 (2 H, m), 7.44 (1 H, m), 7.39 (1 H, m), 7.10 (1 H, m), 6.94 (1 H, m), 6.05 (2 H, s). ^13^C NMR spectrum (DMSO-*d_6_*, 100 MHz p.p.m.): 177.15, 150.63, 143.52,138.59, 135.33, 133.05, 130.91, 129.49, 128.70, 128.01, 188.14, 117.51, 117.13, 115.37, 22.58.

Single crystals were grown *via* vapor diffusion of cyclohexane into a concentrated methanolic solution.

### Refinement

During the course of the refinement, alternate positions for the thiophene sulfur atoms were apparent in the residual electron density maps. Refinement of the site occupancy factors led to values of about 8% for the minor components. Based on these values, a model was constructed for the minor component of each of the thiophene rings using the metrics of the major components as a guide. The pivot atoms (C8 and C13) were assumed to have full occupancy and so were not included in the disorder model. The minor four-atom components (S1', C11', C10' and C9; S2', C16', C15', and C14') were constrained to planarity using FLAT. Using *DFIX*, the S—C bond distances were set to 1.70 Å. Corresponding bond distances of the minor component and major component were set equal using SADI and corresponding thermal parameters were held the same using EADP. All atoms were refined anisotropically with hydrogen atoms in calculated positions using a riding model. With these constraints, the site occupancies of the major components of the methylthiophene and the thiophene refined to 91.6% and 90.3%, respectively. Based on this model, the angles between the mean planes of the major and minor components of the methylthiophene and thiophene are 5.7(1.9)° and 6.6 (9)°.

### Crystal data

**Table:**

C_16_H_12_N_2_S_2_	*F*(000) = 616
*M**_r_* = 296.40	*D*_x_ = 1.371 Mg m^−^^3^
Monoclinic, *P*2_1_/*n*	Mo *K*α radiation, λ = 0.71073 Å
Hall symbol: -P 2yn	Cell parameters from 2556 reflections
*a* = 8.9859 (13) Å	θ = 2.3–24.0°
*b* = 9.1601 (11) Å	µ = 0.36 mm^−^^1^
*c* = 17.476 (3) Å	*T* = 300 K
β = 93.629 (5)°	Plate, clear yellow
*V* = 1435.6 (3) Å^3^	0.80 × 0.30 × 0.10 mm
*Z* = 4	

### Data collection

**Table:**

Bruker SMART X2S benchtop diffractometer	2527 independent reflections
Radiation source: fine-focus sealed tube	1942 reflections with *I* > 2σ(*I*)
graphite	*R*_int_ = 0.033
ω scans	θ_max_ = 25.0°, θ_min_ = 2.5°
Absorption correction: multi-scan (*SADABS*; Bruker, 2008)	*h* = −10→10
*T*_min_ = 0.761, *T*_max_ = 0.965	*k* = −10→10
8857 measured reflections	*l* = −20→20

### Refinement

**Table:**

Refinement on *F*^2^	Primary atom site location: structure-invariant direct methods
Least-squares matrix: full	Secondary atom site location: difference Fourier map
*R*[*F*^2^ > 2σ(*F*^2^)] = 0.040	Hydrogen site location: inferred from neighbouring sites
*wR*(*F*^2^) = 0.103	H atoms treated by a mixture of independent and constrained refinement
*S* = 1.05	*w* = 1/[σ^2^(*F*_o_^2^) + (0.0518*P*)^2^ + 0.2347*P*] where *P* = (*F*_o_^2^ + 2*F*_c_^2^)/3
2527 reflections	(Δ/σ)_max_ < 0.001
210 parameters	Δρ_max_ = 0.18 e Å^−^^3^
26 restraints	Δρ_min_ = −0.25 e Å^−^^3^

### Special details

**Table:**

Geometry. All e.s.d.'s (except the e.s.d. in the dihedral angle between two l.s. planes) are estimated using the full covariance matrix. The cell e.s.d.'s are taken into account individually in the estimation of e.s.d.'s in distances, angles and torsion angles; correlations between e.s.d.'s in cell parameters are only used when they are defined by crystal symmetry. An approximate (isotropic) treatment of cell e.s.d.'s is used for estimating e.s.d.'s involving l.s. planes.
Refinement. Refinement of *F*^2^ against ALL reflections. The weighted *R*-factor *wR* and goodness of fit *S* are based on *F*^2^, conventional *R*-factors *R* are based on *F*, with *F* set to zero for negative *F*^2^. The threshold expression of *F*^2^ > σ(*F*^2^) is used only for calculating *R*-factors(gt) *etc*. and is not relevant to the choice of reflections for refinement. *R*-factors based on *F*^2^ are statistically about twice as large as those based on *F*, and *R*- factors based on ALL data will be even larger.

### Fractional atomic coordinates and isotropic or equivalent isotropic displacement parameters (Å^2^)

**Table:**

	*x*	*y*	*z*	*U*_iso_*/*U*_eq_	Occ. (<1)
S1	0.21859 (8)	0.28769 (8)	0.08330 (3)	0.0467 (2)	0.916 (2)
C9	0.0913 (5)	0.3386 (5)	0.2054 (2)	0.0620 (12)	0.916 (2)
H9	0.0724	0.3488	0.2568	0.074*	0.916 (2)
C10	−0.0188 (4)	0.3558 (5)	0.1464 (2)	0.0648 (10)	0.916 (2)
H10	−0.1168	0.3819	0.1540	0.078*	0.916 (2)
C11	0.0332 (3)	0.3301 (5)	0.07718 (18)	0.0585 (9)	0.916 (2)
H11	−0.0251	0.3349	0.0313	0.070*	0.916 (2)
S1'	0.0789 (14)	0.3656 (15)	0.2202 (7)	0.0467 (2)	0.084 (2)
C9'	0.208 (3)	0.300 (4)	0.1075 (11)	0.0620 (12)	0.084 (2)
H9'	0.2835	0.2677	0.0773	0.074*	0.084 (2)
C11'	−0.017 (3)	0.384 (6)	0.1320 (15)	0.0585 (9)	0.084 (2)
H11'	−0.1150	0.4159	0.1246	0.070*	0.084 (2)
C10'	0.071 (3)	0.344 (6)	0.0747 (16)	0.0648 (10)	0.084 (2)
H10'	0.0434	0.3457	0.0225	0.078*	0.084 (2)
S2	0.18792 (12)	0.42615 (9)	0.47929 (5)	0.0647 (3)	0.903 (2)
C14	0.2441 (4)	0.1896 (4)	0.41297 (19)	0.0411 (8)	0.903 (2)
H14	0.2767	0.1211	0.3785	0.049*	0.903 (2)
C15	0.1692 (5)	0.1519 (4)	0.4778 (3)	0.0510 (8)	0.903 (2)
H15	0.1469	0.0563	0.4907	0.061*	0.903 (2)
C16	0.1329 (4)	0.2674 (5)	0.51922 (17)	0.0549 (10)	0.903 (2)
H16	0.0835	0.2618	0.5643	0.066*	0.903 (2)
S2'	0.2508 (19)	0.1572 (12)	0.3994 (8)	0.0647 (3)	0.097 (2)
C14'	0.187 (3)	0.394 (3)	0.4642 (13)	0.0411 (8)	0.097 (2)
H14'	0.1810	0.4923	0.4762	0.049*	0.097 (2)
C16'	0.146 (5)	0.146 (3)	0.478 (2)	0.0549 (10)	0.097 (2)
H16'	0.1140	0.0590	0.4989	0.066*	0.097 (2)
C15'	0.117 (5)	0.280 (4)	0.5038 (19)	0.0510 (8)	0.097 (2)
H15'	0.0564	0.2969	0.5442	0.061*	0.097 (2)
N1	0.41824 (18)	0.32345 (17)	0.29374 (9)	0.0385 (4)	
N2	0.47184 (19)	0.18925 (19)	0.19147 (9)	0.0453 (4)	
C1	0.5604 (2)	0.2661 (2)	0.31017 (12)	0.0405 (5)	
C2	0.5918 (2)	0.1839 (2)	0.24593 (12)	0.0450 (5)	
C3	0.7282 (3)	0.1124 (3)	0.24430 (15)	0.0606 (7)	
H3	0.749 (3)	0.062 (3)	0.2051 (14)	0.073*	
C4	0.8285 (3)	0.1262 (3)	0.30724 (16)	0.0731 (8)	
H4	0.9203	0.0793	0.3071	0.088*	
C5	0.7950 (3)	0.2087 (3)	0.37082 (16)	0.0671 (7)	
H5	0.8650	0.2161	0.4122	0.081*	
C6	0.6600 (2)	0.2799 (3)	0.37371 (13)	0.0536 (6)	
H6	0.6369	0.3345	0.4162	0.064*	
C7	0.3719 (2)	0.2722 (2)	0.22212 (11)	0.0380 (5)	
C8	0.2290 (2)	0.3058 (2)	0.18189 (11)	0.0398 (5)	
C12	0.3420 (2)	0.4205 (2)	0.34473 (11)	0.0422 (5)	
H12A	0.2690	0.4782	0.3148	0.051*	
H12B	0.4139	0.4870	0.3695	0.051*	
C13	0.2649 (2)	0.3368 (2)	0.40501 (10)	0.0374 (5)	

### Atomic displacement parameters (Å^2^)

**Table:**

	*U*^11^	*U*^22^	*U*^33^	*U*^12^	*U*^13^	*U*^23^
S1	0.0584 (4)	0.0489 (4)	0.0324 (4)	0.0037 (3)	0.0007 (3)	−0.0022 (3)
C9	0.0552 (18)	0.079 (3)	0.053 (2)	0.0069 (17)	0.0129 (16)	−0.0182 (18)
C10	0.0447 (14)	0.078 (3)	0.071 (2)	0.0102 (15)	−0.0007 (15)	−0.0031 (18)
C11	0.0576 (19)	0.0612 (18)	0.0542 (16)	0.0000 (18)	−0.0167 (15)	−0.0041 (14)
S1'	0.0584 (4)	0.0489 (4)	0.0324 (4)	0.0037 (3)	0.0007 (3)	−0.0022 (3)
C9'	0.0552 (18)	0.079 (3)	0.053 (2)	0.0069 (17)	0.0129 (16)	−0.0182 (18)
C11'	0.0576 (19)	0.0612 (18)	0.0542 (16)	0.0000 (18)	−0.0167 (15)	−0.0041 (14)
C10'	0.0447 (14)	0.078 (3)	0.071 (2)	0.0102 (15)	−0.0007 (15)	−0.0031 (18)
S2	0.0900 (6)	0.0564 (6)	0.0515 (5)	−0.0007 (4)	0.0340 (4)	−0.0160 (3)
C14	0.0445 (14)	0.044 (2)	0.0355 (19)	0.0040 (14)	0.0106 (13)	−0.0070 (13)
C15	0.045 (2)	0.0523 (16)	0.0560 (16)	−0.0033 (12)	0.0097 (14)	0.0101 (13)
C16	0.0478 (17)	0.083 (2)	0.0345 (17)	0.0014 (15)	0.0113 (15)	0.0023 (15)
S2'	0.0900 (6)	0.0564 (6)	0.0515 (5)	−0.0007 (4)	0.0340 (4)	−0.0160 (3)
C14'	0.0445 (14)	0.044 (2)	0.0355 (19)	0.0040 (14)	0.0106 (13)	−0.0070 (13)
C16'	0.0478 (17)	0.083 (2)	0.0345 (17)	0.0014 (15)	0.0113 (15)	0.0023 (15)
C15'	0.045 (2)	0.0523 (16)	0.0560 (16)	−0.0033 (12)	0.0097 (14)	0.0101 (13)
N1	0.0465 (9)	0.0396 (9)	0.0300 (9)	−0.0002 (8)	0.0080 (7)	−0.0035 (7)
N2	0.0478 (10)	0.0536 (11)	0.0354 (10)	0.0049 (8)	0.0092 (8)	−0.0070 (8)
C1	0.0421 (11)	0.0424 (12)	0.0375 (12)	−0.0069 (9)	0.0068 (9)	0.0027 (9)
C2	0.0428 (11)	0.0526 (13)	0.0405 (12)	0.0011 (10)	0.0106 (10)	0.0035 (10)
C3	0.0491 (14)	0.0785 (18)	0.0558 (16)	0.0117 (13)	0.0157 (12)	−0.0021 (13)
C4	0.0437 (13)	0.100 (2)	0.076 (2)	0.0125 (14)	0.0076 (14)	0.0171 (17)
C5	0.0483 (14)	0.090 (2)	0.0617 (17)	−0.0087 (13)	−0.0084 (12)	0.0143 (15)
C6	0.0548 (14)	0.0645 (15)	0.0411 (13)	−0.0096 (11)	−0.0009 (11)	0.0010 (11)
C7	0.0458 (11)	0.0380 (11)	0.0309 (11)	−0.0025 (9)	0.0088 (9)	0.0011 (9)
C8	0.0463 (11)	0.0373 (11)	0.0360 (11)	−0.0022 (9)	0.0036 (9)	0.0019 (9)
C12	0.0575 (12)	0.0360 (11)	0.0341 (11)	0.0022 (9)	0.0108 (10)	−0.0061 (9)
C13	0.0398 (10)	0.0423 (12)	0.0303 (10)	0.0048 (9)	0.0046 (9)	−0.0055 (9)

### Geometric parameters (Å, °)

**Table:**

S1—C11	1.708 (3)	C14'—C13	1.388 (17)
S1—C8	1.728 (2)	C14'—C15'	1.42 (2)
C9—C8	1.362 (4)	C14'—H14'	0.9300
C9—C10	1.392 (5)	C16'—C15'	1.345 (19)
C9—H9	0.9300	C16'—H16'	0.9300
C10—C11	1.345 (4)	C15'—H15'	0.9300
C10—H10	0.9300	N1—C7	1.377 (2)
C11—H11	0.9300	N1—C1	1.394 (3)
S1'—C8	1.638 (10)	N1—C12	1.460 (2)
S1'—C11'	1.726 (14)	N2—C7	1.316 (2)
C9'—C8	1.303 (19)	N2—C2	1.393 (3)
C9'—C10'	1.39 (2)	C1—C6	1.387 (3)
C9'—H9'	0.9300	C1—C2	1.395 (3)
C11'—C10'	1.366 (19)	C2—C3	1.392 (3)
C11'—H11'	0.9300	C3—C4	1.383 (4)
C10'—H10'	0.9300	C3—H3	0.86 (2)
S2—C16	1.700 (4)	C4—C5	1.393 (4)
S2—C13	1.7166 (18)	C4—H4	0.9300
C14—C13	1.370 (4)	C5—C6	1.381 (3)
C14—C15	1.397 (4)	C5—H5	0.9300
C14—H14	0.9300	C6—H6	0.9300
C15—C16	1.334 (4)	C7—C8	1.457 (3)
C15—H15	0.9300	C12—C13	1.507 (3)
C16—H16	0.9300	C12—H12A	0.9700
S2'—C13	1.653 (10)	C12—H12B	0.9700
S2'—C16'	1.711 (15)		
			
C11—S1—C8	91.82 (13)	N1—C1—C2	105.49 (18)
C8—C9—C10	114.7 (3)	C3—C2—N2	130.3 (2)
C8—C9—H9	122.6	C3—C2—C1	119.6 (2)
C10—C9—H9	122.6	N2—C2—C1	110.09 (17)
C11—C10—C9	112.0 (3)	C4—C3—C2	118.1 (2)
C11—C10—H10	124.0	C4—C3—H3	121.3 (18)
C9—C10—H10	124.0	C2—C3—H3	120.6 (18)
C10—C11—S1	112.1 (2)	C3—C4—C5	121.4 (2)
C10—C11—H11	123.9	C3—C4—H4	119.3
S1—C11—H11	123.9	C5—C4—H4	119.3
C8—S1'—C11'	92.6 (11)	C6—C5—C4	121.3 (2)
C8—C9'—C10'	118 (2)	C6—C5—H5	119.3
C8—C9'—H9'	121.1	C4—C5—H5	119.3
C10'—C9'—H9'	121.1	C5—C6—C1	116.8 (2)
C10'—C11'—S1'	110.5 (16)	C5—C6—H6	121.6
C10'—C11'—H11'	124.7	C1—C6—H6	121.6
S1'—C11'—H11'	124.7	N2—C7—N1	113.07 (18)
C11'—C10'—C9'	108 (2)	N2—C7—C8	121.92 (18)
C11'—C10'—H10'	125.8	N1—C7—C8	125.00 (17)
C9'—C10'—H10'	125.8	C9'—C8—C9	103.6 (14)
C16—S2—C13	92.54 (14)	C9'—C8—C7	122.6 (13)
C13—C14—C15	113.6 (3)	C9—C8—C7	133.7 (2)
C13—C14—H14	123.2	C9'—C8—S1'	110.7 (13)
C15—C14—H14	123.2	C9—C8—S1'	10.0 (6)
C16—C15—C14	113.1 (3)	C7—C8—S1'	126.6 (5)
C16—C15—H15	123.5	C9'—C8—S1	6.0 (13)
C14—C15—H15	123.5	C9—C8—S1	109.2 (2)
C15—C16—S2	111.6 (2)	C7—C8—S1	116.84 (14)
C15—C16—H16	124.2	S1'—C8—S1	116.5 (5)
S2—C16—H16	124.2	N1—C12—C13	111.81 (16)
C13—S2'—C16'	93.3 (11)	N1—C12—H12A	109.3
C13—C14'—C15'	110.4 (19)	C13—C12—H12A	109.3
C13—C14'—H14'	124.8	N1—C12—H12B	109.3
C15'—C14'—H14'	124.8	C13—C12—H12B	109.3
C15'—C16'—S2'	110.1 (16)	H12A—C12—H12B	107.9
C15'—C16'—H16'	124.9	C14—C13—C14'	102.4 (12)
S2'—C16'—H16'	124.9	C14—C13—C12	130.06 (19)
C16'—C15'—C14'	114 (2)	C14'—C13—C12	127.4 (11)
C16'—C15'—H15'	123.1	C14—C13—S2'	10.1 (5)
C14'—C15'—H15'	123.1	C14'—C13—S2'	112.2 (12)
C7—N1—C1	106.23 (16)	C12—C13—S2'	120.1 (4)
C7—N1—C12	129.49 (17)	C14—C13—S2	109.18 (17)
C1—N1—C12	124.27 (17)	C14'—C13—S2	8.3 (11)
C7—N2—C2	105.12 (16)	C12—C13—S2	120.76 (15)
C6—C1—N1	131.82 (19)	S2'—C13—S2	119.1 (4)
C6—C1—C2	122.7 (2)		

### Hydrogen-bond geometry (Å, °)

**Table:**

*D*—H···*A*	*D*—H	H···*A*	*D*···*A*	*D*—H···*A*
C16—H16···N2^i^	0.93	2.54	3.445 (4)	166

Symmetry codes: (i) *x*−1/2, −*y*+1/2, *z*+1/2.
